# Reconsidering the Differences Between Shame and Guilt

**DOI:** 10.5964/ejop.v14i3.1564

**Published:** 2018-08-31

**Authors:** Maria Miceli, Cristiano Castelfranchi

**Affiliations:** a Institute of Cognitive Sciences and Technologies, National Research Council of Italy, Rome, Italy; Department of Psychology, Webster University Geneva, Geneva, Switzerland; University of Neuchâtel, Neuchâtel, Switzerland

**Keywords:** guilt, shame, self-evaluation, inadequacy, harmfulness, moral emotions, responsibility, self-esteem

## Abstract

Although most researchers maintain that shame and guilt are distinct emotions, the debate on their differences is still open. We aim to show that some of the current distinctions between shame and guilt need to be redrawn, and their adaptive and social implications need to be revisited. We suggest the following distinguishing criteria: the kind of self-evaluation involved (inadequacy versus harmfulness); one’s focus on the perceived discrepancy between actual and ideal self versus one’s focus on the perceived responsibility for one’s fault; and consequently the different domains of self-esteem involved. Although these criteria have been in part suggested or alluded to in the relevant literature, we use and integrate them with each other in a novel way. This allows to better distinguish between shame and guilt, as well as to account for their possible coexistence or the shift from one emotion to the other.

Shame and guilt have much in common: they are self-conscious emotions, implying self-reflection and self-evaluation (e.g., Tangney & Tracy, 2012); they involve negative self-evaluations and feelings of distress elicited by one’s perceived failures or transgressions (e.g., Tangney, Stuewig, & Mashek, 2007); they strongly correlate with each other (e.g., Ferguson & Crowley, 1997; Harder, 1995), and often coexist (Eisenberg, 2000; Lewis, 1971). However, most researchers maintain that shame and guilt are distinguishable from each other, and that their differences matter. While agreeing with this general statement, we disagree with some of the criteria used for distinguishing between them, and with the prevailing negative view of shame which, in comparison with guilt, often plays the role of the “ugly” and anti-social emotion (e.g., Tangney & Tracy, 2012).

Before discussing the most common criteria used for distinguishing between shame and guilt, and then suggesting our own criteria, we need to explain why it is important to distinguish between these emotions. In so doing, we will also outline the core of our proposal.

As widely acknowledged, emotions accomplish both an informative function about our relationship with the environment, by signaling the (prospective or actual) failure or attainment of our goals (e.g., Damasio, 1994; Keltner & Ekman, 2000; Lazarus, 1991) and a motivational function (e.g., Frijda, 1986; Plutchik, 1984), by triggering goals aimed at favoring the attainment, or avoiding the failure, of the desired states of affairs. Therefore it is generally adaptive to understand what the experience of an emotion is “telling” us.

Both shame and guilt are “self-critical” emotions. However, self-criticism may take different self-evaluative forms: on the one hand, people may view themselves as ugly, stupid, handicapped, or morally defective—in a word, *lacking* (in physical attractiveness, intelligence, skills, moral worth, and so on); on the other hand, they may view themselves as wicked, unjust, sinful—that is, *endowed with the power to violate norms and thwart others’ goals, and willing (or inclined) to do so*.

We suggest that it is adaptive to have differentiated emotional responses to different forms of self-criticism. As also pointed out by Tangney, Miller, Flicker, & Barlow (1996, p. 1256), “to the extent that emotions inform and foster change, one might expect humans to develop particularly well-articulated affective responses to negative events”. Thus, it is worthwhile to explore what kind of information is conveyed and what kind of change is fostered by each self-critical emotion.

As we will argue, guilt implies a negative *moral* self-evaluation. Without attempting to give a definition of *moral*, we suggest that a necessary condition for regarding an evaluation as moral is that it should concern someone’s behavior, goals, beliefs or traits for which (s)he is regarded as responsible. The evaluation will be positive or negative depending on the beneficial or harmful quality ascribed to such behavior, goal, and so on. Guilt is indeed concerned with one’s responsibility for a harmful attitude or behavior.

By contrast, shame implies a nonmoral negative self-evaluation. Note that “nonmoral” is not synonym to “immoral”. By “nonmoral” we mean that shame is not focused on responsibility issues. As we shall see, it is rather concerned with a perceived discrepancy between one’s actual and one’s ideal self. In fact, one may feel ashamed of one’s ugliness, disability, or any other flaw for which (s)he is not responsible. However, one may also feel ashamed (rather than guilty) of a responsible fault. We will suggest that even when a responsible fault is at stake, ashamed people do not focus on responsibility issues, but on the disappointing fact that such fault reveals their defectiveness with regard to their ideal self. To the extent that shame can make people care about the social order, it can be said to be a moral emotion (e.g., Rozin, Lowery, Imada, & Haidt, 1999). However, to the extent that this care is motivated by a self-focused concern (i.e., building one’s aspired-to identity), shame is not properly moral, in the sense we have outlined above. According to Rozin et al. (1999, p. 574), both shame and guilt “involve ongoing assessments of the moral worth and fit of the individual self within a community”. We also suggest that shame is concerned with self-worth. But, first, it is concerned with both moral and nonmoral self-worth; second, when moral self-worth is at stake, what matters to the ashamed person is *not* his or her responsibility for the fault, but how this fault impacts on his or her ideal self.

Shame implies perceived *lack of power* to meet the standards of one’s ideal self, whereas guilt implies perceived *power and willingness to be harmful*, that is, to violate the standards of one’s moral self. These differences have important motivational consequences, both positive and negative: whereas guilt is likely to motivate either reparative or self-punitive behavior, shame is likely to motivate either withdrawal or increased efforts in building one’s aspired-to identity. More generally, distinguishing between shame and guilt has significant implications for basic research on human emotions as well as for clinical treatment, by helping understand if and how these emotions relate to a variety of phenomena, such as empathic concerns, narcissism, self-destructive behavior, and moral compliance.

As we will try to show, the distinguishing criteria suggested in the relevant literature do not satisfactorily account for the differences between shame and guilt, whereas our own criteria allow to better identify the respective features of the two emotions.

## Criteria Used for Distinguishing Between Shame and Guilt

We will group the most widespread distinguishing criteria into three categories: the kinds of failure or transgression that elicit these emotions; the action tendencies typically triggered by shame versus guilt; and the ascription of the fault to one’s self versus one’s behavior.

In addition, we will consider a number of elements which are viewed as indicative of remarkable differences between shame and guilt in terms of their adaptive value: the cognitive states, as well as other feelings, that are associated with shame versus guilt; and the psychopathological symptoms related to shame-proneness versus guilt-proneness.

### Kinds of Failure or Transgression

Three possible kinds of fault have been identified: the *public* versus *private* experience of the fault; its *proscriptive* versus *prescriptive* nature; and the *moral* versus the *both moral and nonmoral* nature of the fault.

#### Public Versus Private

According to this distinction, shame is elicited by public faults, and (one’s fear of) others’ negative evaluations, whereas guilt is a private feeling, elicited by one’s own negative self-evaluation (e.g., Benedict, 1946; Combs, Campbell, Jackson, & Smith, 2010; Wallbott & Scherer, 1995).

The public versus private criterion has already been questioned, both theoretically and empirically. For instance, Creighton (1990, p. 282) has objected that “the internal/external criterion cannot be used to distinguish guilt from shame, since at some point in the developmental process both are internalized”. Empirical research has indeed shown that both emotions can be experienced either publicly or privately (e.g., Tangney et al., 1996).

Still, this does not rule out that, when experienced privately, shame might imply either thinking that one’s fault *can become* public or *imagining* a judging audience. As often suggested, shame is more likely than guilt to imply a feeling of exposure to a judging audience (e.g., M. Lewis, 1992). According to Kaufman (1996, p. 28), “to feel shame is to feel seen, acutely diminished”—which is also supported by the person’s typical wish to disappear and tendency to hide (e.g., Darwin, 1872/1965; H. B. Lewis, 1971; M. Lewis, 2008). In fact public exposure of one’s faults has been found to be associated more with shame than with guilt (Smith, Webster, Parrott, & Eyre, 2002).

However, if one assumes that shame is elicited by (actual, or expected, or imagined) external sanctions, one might draw the inference that this emotion coincides with a mere fear of others’ disapproval, and that it can be experienced without evaluating oneself negatively—which is indeed stated by some authors (e.g., Ausubel, 1955; Calhoun, 2004; Wollheim, 1999).

We question that shame coincides with being afraid of others’ disapproval. One can be afraid of other people’s negative evaluations while feeling no shame. For instance, a son who fails to live up to the standards set by his parents can be afraid to disappoint them or to be punished by them, and at the same time experience no shame.

In agreement with a number of other authors (e.g., Deonna, Rodogno, & Teroni, 2012; Tangney & Tracy, 2012; Taylor, 1985), we suggest that *shame (as well as guilt) implies a negative self-evaluation against one’s own standards*. Although personal standards result from the internalization of social ones, they are not necessarily absorbed in the same form as they are socially conveyed. Internalization involves developing a personalized interpretation of social standards (e.g., Baumeister & Muraven, 1996; Lawrence & Heinze, 1997), consisting not only in the combination of existing social elements into new configurations, but also in the selective acceptance of some elements and rejection of others (e.g., Grusec & Goodnow, 1994).

In our view, if a negative evaluation comes from outside, one has to *share* it in order to feel ashamed (as well as guilty). In fact, others’ evaluation and one’s self-evaluation may diverge, either because one may not share the evaluative standards of one’s “judges” or because, while sharing their standards, one may believe that their evaluations are *mistaken* in that no actual fault has been shown according to those standards.

We suggest that in both the above cases the disapproved person will not experience shame (and certainly not guilt), but other possible feelings—fear of the consequences of the negative evaluation; disappointment and resentment for having received an “undeserved” evaluation; helplessness, elicited by the perceived inability to modify others’ judgment; or, most notably, embarrassment, which implies no necessary negative self-evaluation (e.g., Sabini & Silver, 1997), but mere *discomfort at exposure* (Miller, 1985; Nussbaum, 2004). Embarrassment is supposed to be more focused on one’s *self-presentation* (Klass, 1990), rather than on one’s self-evaluation. As shown by Tangney et al. (1996, p. 1260), whereas shame could be felt in private, “embarrassment was almost universally a public phenomenon”.

We also question that for feeling ashamed one should think that one’s fault can become public. One can even *be certain that nobody will ever know* about one’s own fault, and still experience shame. In the same vein, one does not need to imagine a judging audience. As claimed by Deonna et al. (2012), although a real or imagined audience is *typical* of shame, it does not need to be *constitutive* of the emotion. Of course, one learns socially what is “shameful”, but one also learns socially what is threatening, or disappointing, or saddening—which does not imply the necessary involvement of an audience in the experience of fear, disappointment, or sadness.

What we have said so far, however, by no means excludes that shame involves “to feel seen”. Even without any real or imagined audience, one can feel seen by one’s self in the role of self-evaluator, and experience shame. In Kaufman’s (1996, p. 6) words, “[o]nly the self need watch the self and only the self need shame the self”.

#### Proscriptive Versus Prescriptive

According to Sheikh and Janoff-Bulman (2010), proscriptive violations (doing something one should not do) would elicit shame, whereas prescriptive violations (not doing something one should do) would elicit guilt.

However, a proscriptive violation (e.g., lying) may elicit guilt, and a prescriptive violation (not reciprocating a favor) may elicit shame. As found by Keltner and Buswell (1996), the most frequent antecedents of shame were: poor performance, hurting others’ feelings, failing to meet either others’ or one’s own expectations, and showing a role-inappropriate behavior. The most frequent antecedents of guilt were: failures at duties, lying, neglecting a dear one, breaking a diet, and cheating. Both proscriptive and prescriptive violations seem capable of eliciting either shame or guilt.

#### Moral Faults Versus Both Moral and Nonmoral Faults

According to some authors (e.g., Sabini & Silver, 1997; Smith et al., 2002), guilt is elicited by moral transgressions, implying those faults one is held responsible for, whereas shame also includes nonmoral faults, that is, those attributed to flaws of character, incompetence or physical inadequacies for which the person is not held responsible. (For a discussion of perceived responsibility and guilt, see further on, “Kinds of Negative Evaluation”, and “Is Perceived Responsibility Necessary for Feeling Guilty?”) But, when does a *moral* fault elicit shame rather than guilt?

Smith et al. (2002) suggest that guilt is associated with private moral faults, whereas shame is more closely linked to public ones. However the public-versus-private distinction is not decisive in that shame can be felt even privately. Sabini and Silver (1997) suggest that guilt pertains to the domain of moral evaluation, which is grounded in responsibility. However, they claim that, as long as a moral fault involves a connection with the self by indicating a flaw in one’s character, it also involves shame. They view shameless guilt as an “anemic” feeling. They even cast doubt on whether guilt is a distinct feeling, and suggest that it is “an umbrella that collects under itself a broad range of feelings having to do with transgressions” (p. 3), including shame. Shame can be also elicited by nonmoral faults because it is “a fundamentally aesthetic response to our judgments of our character” (p. 12), that is, to evaluations of ourselves as ugly, repulsive, or inferior, even though we feel unable to be otherwise. In fact, self-worth includes both the moral and nonmoral (or aesthetic) domain: we can be ashamed of our wrongdoings as well as of flaws that are not our fault.

Thus, in Sabini and Silver’s (1997) view, shame takes the lion’s share, while (shameless) guilt is an “anemic” feeling. We will go back to this point when we discuss our own distinguishing criteria. (See “Can Perceived Responsibility Concern the Self Rather Than Specific Behaviors?”, and “Is Perceived Responsibility a Sufficient Cognitive Component of Guilt?”)

### Kinds of Action Tendencies

Guilt has been found to lead to repair action tendencies, such as apologizing, amending, and undoing, whereas shame appears to favor withdrawal and escape behaviors, as well as hostile and self-defensive reactions (e.g., Tangney & Dearing, 2002). On the ground of such findings, shame has been stigmatized as an ugly and anti-social emotion, whereas guilt has been viewed as a moral and prosocial emotion (e.g., Tangney & Stuewig, 2004; Tangney et al., 2007).

However, the relationship between emotions and action tendencies is not so straightforward as one might expect. As shown by Schwarz and Clore (2007), emotions present a tenuous causal link with specific action tendencies, whereas their immediate effects are more mental than behavioral. Action tendencies depend on contextual demands more than on specific emotion categories (e.g., Barrett, 2006).

No doubt, guilty people are likely to wish that they had not committed the wrongdoing, and to feel the need to make amends (e.g., Gino & Pierce, 2009; Iyer, Leach, & Pedersen, 2004), but these motivations emerge *on condition that people consciously recognize their responsibility*. Self-defensive maneuvers are not monopoly of shame. People also defend themselves from guilt (Miceli & Castelfranchi, 1998) by denying the intentionality of the misdeed (e.g., Baumeister & Wotman, 1992), downplaying its negative consequences (Stillwell & Baumeister, 1997), and derogating their victims (Zechmeister & Romero, 2002) so as to represent the wrongdoing as “deserved” by them.

When responsibility is consciously acknowledged, guilt does not necessarily elicit prosocial reparative actions. Acts of self-punishment have been found to be quite common (Inbar, Pizarro, Gilovich, & Ariely, 2013; Nelissen & Zeelenberg, 2009), and to be associated with non-prosocial and destructive tendencies (Fedewa, Burns, & Gomez, 2005).

Turning to shame, although withdrawal is among the action tendencies it elicits, some aspects of withdrawal might be viewed in a prosocial light, by communicating surrender and appeasement (e.g., Barrett, 1995; Castelfranchi & Poggi, 1990; Fessler, 2007; Izard, 1977; Keltner & Harker, 1998). Displays of shame show similarities with the submissive and appeasement behavior of nonhuman animals (Gruenwald, Dickerson, & Kemeny, 2007), and favor reconciliation (Keltner & Harker, 1998) and others’ empathy and forgiveness (Keltner, Young, Heerey, Oemig, & Monarch, 1998).

Shame has been attributed the function to promote social cohesion through the individual’s compliance with social values and expectations (e.g., Barrett, 1995; Deonna et al., 2012; Fessler, 1999; Izard, 1977). If shame were to motivate plain conformity (Gilbert, 2003) so as to avoid external sanctions, this function might be viewed in a negative light: shame would only favor a self-interested and unprincipled compliance. However, such charges lose their force if, when external sanctions are at stake, shame is experienced only if one shares the standards of one’s judges, and believes that their evaluations are correct. Shame is *not caused*, but (often) *triggered* by external sanctions (Deonna et al., 2012), thereby favoring one’s self-evaluation and sensitization to social values—provided one shares them.

Shame has been found to be associated not only with withdrawal, but also with an “undo” desire, which showed high scores for guilt as well (Frijda, Kuipers, & Ter Schure, 1989), with healthy behavior (Harris & Darby, 2009), inhibition of wrongdoings (Ferguson, Edmondson, & Gerity, 2000), prosocial behavior (de Hooge, Breugelmans, & Zeelenberg, 2008), and motivation for self-change (Lickel, Kushlev, Savalei, Matta, & Schmader, 2014). De Hooge, Zeelenberg, and Breugelmans (2010, 2011) found that shame activated both a motivation to *restore* one’s threatened self-image, and a *protect* motive to avoid further damage to one’s self-image. Both motives, when acting jointly, induced approach behaviors such as developing new skills or redoing one’s performance. However, when restoration of the threatened self was perceived as too difficult, the restore motive declined, and approach behaviors diminished accordingly, whereas the relative strength of the protect motive increased, leading to withdrawal behaviors.^i^

Gausel, Vignoles, and Leach (2016) have addressed the “paradox” of shame, that is, the coexistence of self-defensive and prosocial responses activated by perceived moral failure. They show that moral failure can cause two distinct appraisals: the appraisal of “specific self-defect”, that implies a concern for one’s own *self*-image and leads to felt shame, which in turn predicts prosocial responses aimed at restoring the self-image; and the appraisal of “concern for social condemnation”, that focuses on the risk to one’s *social* image, and leads to feelings of *rejection* (rather than shame proper), which in turn predict self-defensive avoidance. By distinguishing social-image concerns from self-image concerns, and the effects of felt rejection from those of felt shame, these findings suggest that shame proper is unrelated to self-defensive action tendencies.

Therefore, based on the literature reviewed above, it seems unwarranted to conclude that shame is characterized by self-defensive action tendencies, and guilt by prosocial ones. Both emotions can elicit both prosocial and self-defensive behavior.

### Self Versus Behavior

This distinction does not focus either on the motivational implications of shame versus guilt or on the different kinds of fault which would provoke these emotions. In fact, shame and guilt can be elicited by the same failure or transgression (e.g., a poor performance). According to the self-versus-behavior view, what matters is whether the fault is ascribed to the self or is circumscribed to one’s behavior: If it is ascribed to the self, which is supposed to imply a global negative self-evaluation (“I *am* a bad, inadequate *person*”), the emotional response will be of shame. If one’s own negative evaluation is circumscribed to one’s behavior (“I *did* a bad *thing*”), it will give rise to guilt (e.g., Lewis, 1971; Tangney & Dearing, 2002). Both shame and guilt imply internal attributions. However shame “is generated when people blame the stable, uncontrollable self for failure, whereas guilt occurs from blaming an unstable, controllable action taken by the self” (Tracy & Robins, 2006, p. 1349).

The self-versus-behavior view is presently considered the mainstream one (see e.g., Fontaine, Luyten, Estas, & Corveleyn, 2004; Gausel, 2012). However, we suggest that *guilt* is not necessarily circumscribed to one’s behavior*—it may imply blaming one’s self*; and shame does not necessarily imply a *global* negative self-view.

One may feel guilty for *being* a cowardly (or selfish, inconsiderate, lazy) *person*. Even when starting from a specific wrongdoing, one may generalize one’s negative evaluation to the self: “Because I did something bad, I *am* bad”. It has been suggested that the behavior-to-self generalization implies a “fusion” between guilt and shame (e.g., Tangney, Burggraf, & Wagner, 1995). However, as we will further discuss (see “Can Perceived Responsibility Concern the Self Rather Than Specific Behaviors?”), this “fusion” assumption is questionable.

Shame, in turn, does not necessarily imply a *global* negative self-view. It may be confined to a specific self-defect (e.g., Gausel et al., 2016). The possibility of “state-specific feelings of shame” is admitted by Tangney and Tracy (2012, p. 454), who even observe that “the vast majority of people’s quotidian transgressions and errors do not warrant a shameful, global condemnation of the self”. We ask: If shame may imply a negative self-evaluation which does not involve the whole self, why build a model of shame in terms of global self-blame?

Although the self-versus-behavior view has received wide empirical support (e.g., Tangney & Dearing, 2002; Tangney et al., 2007; Tracy & Robins, 2006), the Test of Self-Conscious Affect, or TOSCA (Tangney, Dearing, Wagner, & Gramzow, 2000; Tangney, Wagner, & Gramzow, 1989), which is used in many studies addressing shame-proneness versus guilt-proneness, *relies* on the self/behavior distinction. The TOSCA items designed to assess shame-proneness mainly refer to global negative evaluations of the self, and to the tendency to withdraw; whereas those designed to assess guilt-proneness focus on specific negative evaluations of one’s behavior, and the tendency to make amends. Moreover, the TOSCA shame scale has been found to correlate strongly with *both* chronic shame and chronic guilt, whereas the TOSCA guilt scale related to reparative action tendencies, but showed no (or weak) relation to guilt *feelings* (Fontaine, Luyten, De Boeck, & Corveleyn, 2001; Giner-Sorolla, Piazza, & Espinosa, 2011; Luyten, Fontaine, & Corveleyn, 2002). Thus, the self-versus-behavior view appears to be questionable.

### Cognitive, Emotional, and Psychopathological Implications of Shame and Guilt

Shame, especially shame-proneness, is often associated with anger (e.g., Bear, Uribe-Zarain, Manning, & Shiomi, 2009; Tangney & Dearing, 2002). However, anger has been found to be associated with shame-related withdrawal action tendencies, but not with shame-related negative self-evaluations (Cohen, Wolf, Panter, & Insko, 2011). As shame does not necessarily induce withdrawal, its link with anger could be less robust than often assumed.

The relationship between shame and anger has been explained in terms of a self-defensive reaction: to defend from self-blame, one would *try not to take responsibility* for one’s own fault by externalizing it (e.g., Tangney & Tracy, 2012). The hostility that is initially directed inward would be redirected outward.

We are not persuaded by this explanation. We view shame-related anger as a more immediate and “primitive” reaction to frustration – namely, the frustration of one’s self-esteem – and its *causal* attribution to another agent, regardless of any responsibility issue. In contrast with the most common view (e.g., Averill, 1982; Weiner, 1985), we in fact suggest that an attribution of responsibility is *not* a necessary antecedent of an angry reaction.^ii^ In order to feel anger at somebody it is sufficient that one believes that the latter has caused the frustration of one’s own goals (Batson et al., 2007; Dubreuil, 2015). This causal attribution is different from an attribution of responsibility proper (see, e.g., Smith & Lazarus, 1993), which includes controllability and intentionality considerations.

Guilt has been found to be unrelated to anger, and negatively correlated with the externalization of blame (e.g., Tangney & Tracy, 2012). Actually, guilt *implies* viewing oneself as responsible for the fault. Therefore, an angry outward reaction is out of place (provided one does not try to defend against guilt).

Guilt is also associated with perspective-taking and empathic concerns, that in turn favor prosocial behavior (e.g., Batson, 1991), whereas shame seems to interfere with empathy, and to be associated with narcissistic concerns and personal distress responses (Gilligan, 2003; Tangney & Dearing, 2002). This difference between guilt and shame has been explained in terms of the self-versus-behavior focus: “[P]eople experiencing guilt are relatively free of the egocentric, self-involved process underlying shame. Instead, their focus on a specific behavior is likely to highlight the consequences of that behavior for distressed others, further facilitating an empathic response” (Tangney & Tracy, 2012, p. 450).

By contrast, we suggest that the reason why guilt is associated with perspective-taking and empathic concern lies in its focus on one’s *responsibility* for the fault. As long as guilt implies the conviction of having responsibly broken a norm (which is supposed to promote and defend the collective welfare) or having injured someone (Izard, 1977; Lazarus, 1991; Lewis, 1971; McGraw, 1987; Roseman & Evdokas, 2004; Smith & Ellsworth, 1985), it does not merely favor, but *requires* perspective-taking (e.g., Hoffman, 2000). For feeling guilty, one must feel responsible for one’s faults; and for feeling responsible one has to consider others’ needs and concerns, and see the consequences of one’s own behavior and attitudes through their eyes.

We also suggest that the reason why shame is not associated with perspective-taking and empathic concern does not lie in the focus on oneself as a bad person as opposed to one’s own behavior. As we will further discuss (see “Our Distinguishing Criteria”, and the subsection “Is Perceived Non-Responsibility a Necessary Cognitive Component of Shame?”), the crucial aspect is that *responsibility is not an issue for shame*.

Turning to the psychopathological symptoms associated with shame-proneness versus guilt-proneness, there is a wide consensus on the dysfunctional consequences of shame-proneness (depression, generalized anxiety disorder, low self-esteem), whereas there is less consensus on the psychopathological implications of guilt-proneness (e.g., Tangney & Tracy, 2012). However, it is unclear whether the findings on shame-proneness can be generalized to state-specific feelings of shame (e.g., de Hooge et al., 2008; Deonna et al., 2012). Moreover, even sticking to shame-proneness, some measures used to assess it may be unable to detect adaptive aspects of shame, such as appeasement behavior, compliance with shared social standards, and attempts at skill acquisition. This in particular applies to the TOSCA shame scale, focused as it is on global negative self-evaluations and withdrawal action tendencies. Conversely, TOSCA guilt, focused as it is on reparative action tendencies and negative evaluations of specific behaviors, may be unable to detect maladaptive aspects of guilt, such as obsessive rumination, self-punishment, and excessive self-criticism, which are related to depression, anxiety, obsessive-compulsive disorders, and psychoticism (e.g., Harder, 1995; O’Connor, Berry, & Weiss, 1999). TOSCA suffers indeed from construct underrepresentation (Ferguson & Stegge, 1998; Luyten et al., 2002; O’Connor et al., 1999).

Therefore, it is unwarranted to conclude either that shame is an “ugly” and dysfunctional emotion or that guilt is constructive and adaptive. We suggest that many dysfunctional consequences that have been ascribed to shame depend on a *global negative self-view*, which *may* be associated with shame as well as guilt, when negative self-evaluations are generalized to the whole self.

## Our Distinguishing Criteria

As discussed so far, shame and guilt do not seem to be distinguishable from each other according to such criteria as the kinds of fault that elicit these emotions, their action tendencies, and their adaptive versus maladaptive implications. We have also questioned that the self-versus-behavior criterion adequately distinguishes shame from guilt. We do not see why guilt should be only felt about one’s own behavior, and will argue that it can also be felt about who the person *is*; on the other hand, we contend that shame is *not* necessarily focused on the self *as a whole*.

We are going to suggest two criteria which in our view allow to better distinguish shame from guilt. The first criterion regards the different *kinds of negative self-evaluation* implied by these emotions: Whereas shame implies a self-evaluation of *inadequacy*, guilt implies a self-evaluation of *harmfulness*. This is a new criterion. Although a sense of personal inadequacy or powerlessness has been typically associated with shame (e.g., Tangney, 1999), and one’s own perceived harmfulness has been associated with guilt (e.g., Baumeister, Stillwell, & Heatherton, 1994), the kind of negative self-evaluation involved in shame versus guilt has, to our knowledge, never been raised to the dignity of a distinguishing criterion.

Our second criterion consists in the different *focuses* typical of these emotions: Whereas shame is concerned with the discrepancy between a negative self-evaluation and the positive, desired one, guilt is concerned with responsibility for one’s faults. This criterion is not new in itself. That shame is triggered by the perceived discrepancy between one’s actual self and one’s ideal self has been already suggested (e.g., Higgins, 1987; Lazarus, 1991). In the same vein, responsibility has already been considered to be constitutive of the appraisal for guilt (e.g., Lazarus, 1991; Roseman & Evdokas, 2004; Smith & Ellsworth, 1985). Attributional models of guilt (e.g., Tracy & Robins, 2006) also seem to acknowledge the crucial role played in guilt by perceived responsibility, by relating guilt to internal, unstable, and controllable attributions for negative outcomes.

However, the role of responsibility in shame has so far remained obscure. As pointed out by some authors (e.g., Sabini & Silver, 1997; Smith et al., 2002), shame can be elicited by either responsible or non-responsible faults. But how is it possible that a responsible fault elicits shame (rather than guilt)?

One might endorse the attributional view, which relates shame to internal, stable, and uncontrollable attributions (e.g., Tracy & Robins, 2006), and suggest that, independent of whether ashamed people are actually responsible for the fault, they *perceive themselves as non-responsible*, precisely because they trace back the fault to stable and uncontrollable causes. However, we are going to question the attributional account of shame (see next section), as well as the implication that the ashamed person should necessarily perceive themself as *non-responsible* for the fault. (See “Is Perceived Non-Responsibility a Necessary Cognitive Component of Shame?”)

### Kinds of Negative Self-Evaluation: Inadequacy Versus Harmfulness

Evaluations can be defined as beliefs about “what is good/bad for what” (Miceli & Castelfranchi, 2000). They imply the assignment of a (positive or negative) value to an entity, event, or world state *x* in that the latter is viewed as a good or bad means for some (class of) goal(s). Evaluative beliefs can be either explicit (say, “This knife is good for slicing food”) or implicit (say, “This knife is sharp”). The positivity or negativity of the evaluation is contingent on the specific goal for which *x* is viewed as a means. For instance, a sharp knife is “good” for a cook’s goal of slicing food, whereas it is “bad” for a child’s goal of putting butter on the bread.

Negative (self-)evaluations can be of two kinds—harmfulness versus inadequacy^iii^ (Miceli & Castelfranchi, 2000). A knife can be evaluated as “bad” either because it is blunt, and *unable* to cut properly, or because it is too sharp, and *able* to hurt. When appraised as inadequate, *x* is regarded as possessing *insufficient* power (properties, skills, attitudes) with respect to some goal (in our example, the goal of cutting something); when appraised as harmful, *x* is regarded as possessing sufficient power to attain a *negative* goal, that is, to realize a world state not-*p* which is the opposite of someone’s goal *p* (in our example, the goal of physical safety).^iv^

Whenever the power to thwart someone’s goals is associated with the corresponding *goal* or, at least, with the *power to prevent* such harm, (self-)evaluations of harmfulness are strictly linked to responsibility issues. In fact, to regard oneself as responsible for something, one should believe that: (a) one caused it (causal responsibility); and (b) one had the goal to cause it (goal responsibility), or at least (c) one had the power to prevent it (avoidance responsibility), but omitted to do so (Miceli, 1992; Weiner, 1995).

Feeling guilty implies perceiving oneself as a wrongdoer, which entails a self-evaluation of *responsible harmfulness,* that is, perceiving oneself as responsible for one’s harmful behavior or attitude.^v^ When self-evaluations concern a lack of power which is perceived to be beyond one’s own control, one cannot feel guilty. People cannot feel guilty for their ugliness or handicaps—unless they view themselves as capable of self-improvement, and therefore *responsible* for not trying to self-improve. By contrast, they can feel ashamed of their ugliness or handicaps, because a self-evaluation of mere *inadequacy* is sufficient (as well as necessary) for feeling shame.

So far, our distinguishing criteria seem to overlap with those suggested by attributional models of shame and guilt (e.g., Tracy & Robins, 2006). However, according to those models, guilt implies internal, unstable, and controllable attributions, whereas shame is qualified by internal, *stable*, and *uncontrollable* attributions.

While endorsing the attributional model of guilt, we view shame as less cognitively sophisticated, and suggest that the only necessary and sufficient attribution is the *internal* one. The self-attributed inadequacy is *not necessarily* perceived as stable and uncontrollable. It is sufficient that people focus on *the discrepancy between a negative self-evaluation and the positive one they would like to have*. Of course, a further attributional search can be performed: *If* one’s fault is traced back to uncontrollable and stable causes, shame will be associated with helplessness and hopelessness. Conversely, if one’s fault is attributed to unstable and controllable causes, guilty feelings can arise—which may account for the frequent coexistence of shame and guilt, or more plausibly, the *shift* from one emotion to the other. But all of those consequences are *possible* implications of a further attributional search.

The issue of perceived responsibility, however, needs to be better specified. We have to establish if it is actually necessary as well as if it is sufficient for experiencing guilt. We also need to explain how perceived responsibility can involve the self, without necessarily implying a fusion between guilt and shame. Finally, we should clarify why perceived responsibility is *not* involved in shame even when it concerns *moral* faults.

### Is Perceived Responsibility Necessary for Feeling Guilty?

Some authors consider responsibility unnecessary for feeling guilty (e.g., Baumeister et al., 1994; Berndsen & Manstead, 2007). In various instances of guilt, responsibility indeed seems to play no or little role. However, before discussing these counterexamples, two clarifications are in order.

First, perceived responsibility does not necessarily coincide with actual responsibility—it may even be grounded on irrational beliefs (e.g., the thought that one may cause harm to others by simply wishing it). Unsurprisingly, innocent rape victims often feel co-responsible for the rape because they think they provoked it through their appearance or behavior, or did not do everything possible to prevent the attack (e.g., Janoff-Bulman, 1979; Meyer & Taylor, 1986). Second, we admit that an appraisal of personal responsibility proper – implying both causal and either goal or avoidance responsibility – is not a necessary *antecedent* of guilt (see, e.g., Berndsen & Manstead, 2007). For eliciting a first “pang” of guilt, it may suffice to assume one’s *causal* responsibility for a harm (e.g., Frijda, 1993). Developmentally speaking, perceiving oneself as a cause of another’s suffering is the core of guilt (e.g., Zahn-Waxler & Kochanska, 1990). We suggest that an assumption of responsibility proper is a necessary *constituent* of guilt as a full-blown emotion, which may result from further elaboration of an initial, cognitively “poorer” emotional experience. However, once this cognitive elaboration has taken place, an assumption of responsibility proper is a necessary ingredient of guilt. If people are able to rule out both their goal responsibility and their avoidance responsibility for a transgression, they can get rid of the feeling, even when their causal role remains undisputed. Unsurprisingly, therapeutic treatments aimed at removing feelings of guilt often focus on challenging the clients’ assumptions of responsibility (e.g., Lamb, 1986).

Let us now discuss a few cases of guilt “without responsibility”. People who are over-rewarded tend to feel guilty when their advantage causes another’s disadvantage (e.g., Hegtvedt & Killian, 1999). Even though they can view their advantage as *undeserved*, this condition seems insufficient to imply perceived responsibility for the inequity as long as they believe they neither did anything to cause it nor had the goal to cause it or the power to prevent it. However, they are now *responsibly accepting* an inequitable situation. They can feel responsible for *not* refusing the advantage, not trying to re-distribute what is undeserved, or not challenging the standards followed by the bestowers of rewards. Responsibility can regard not only acts of commission but also omissions.

Another classical example of guilt without responsibility is survivor guilt (e.g., Brockner, Davy, & Carter, 1985). In many such instances, people acknowledge that their guilty feelings are “illogical”, and still declare that they *feel responsible*. How can one feel responsible for not sharing others’ misfortunes?

To start with, after an event people tend to overestimate their preexisting predictive capabilities—showing the well-known hindsight bias (e.g., Fischhoff, 2003)—as well as their control over the situation. Therefore, they may start a chain of counterfactual thoughts about what they could and should have (not) done (for instance, “I scrambled over others to escape”; or “I thought only of myself, without trying to save others”).

Second, let us suppose that a survivor fails to find some far-fetched responsibility to have (not) done something, and reaches the conclusion that being alive is a matter of luck. Luck is often (irrationally) viewed in terms of a zero-sum game—to have good luck implies depriving another of it. Although the survivor is not responsible for this deprivation (the “responsible” one is Fate or God), (s)he is likely to feel happy that (s)he has escaped death, which somehow implies being *happy that another died*. The survivor can feel responsible for having experienced such a despicable feeling, which may be viewed as betraying a disposition to harm others as a means for pursuing one’s own interest. Or, even without self-ascribing the wretched feeling of *schadenfreude*, the survivor may feel guilty for being happy while others are grieving over their losses, and view one’s own happiness as a sign of self-centeredness and hard-heartedness (e.g., Jäger & Bartsch, 2006).

### Can Perceived Responsibility Concern the Self Rather Than Specific Behaviors?

The negative evaluation of one’s behavior can undergo generalization to the self and elicit guilt, without a necessary fusion with shame. Moreover, the mere reflection on one’s own “character”, independent of an actual wrongdoing, may elicit guilt. However, our emphasis on perceived responsibility seems to conflict with these claims. How can one perceive oneself as *responsible* for one’s own traits, rather than for specific wrong*doings*? Isn’t character something one cannot choose and be responsible for (e.g., Sabini & Silver, 1997)?

Many traits *can* be viewed as liable to change through one’s own effort, rather than as stable and uncontrollable features of the self. Much depends on the perspective and implicit theories of the person. For instance, intelligence can be viewed either as a fixed entity or as a malleable quality (e.g., Dweck, 1999). Conversely, willingness (including effort and persistence) is not necessarily viewed as unstable and controllable. It may acquire an “ability” connotation—think of the notion of “weakness of will” (e.g., Davidson, 1980). Persistence can also be viewed as a stable disposition (e.g., Hancock & Szalma, 2008).

Therefore, we suggest that one can feel guilty for one’s negative traits, provided one *feels responsible* for them. And one feels responsible for them if one believes to be *capable of modifying* them – thereby preventing the (potential or actual) harm they engender – *while omitting to do so*. We acknowledge that guilt relates to the self as an *agent*, but this is different from assuming that guilt is exclusively concerned either with one’s *behavior* or with how one’s *actual* behavior reflects on the self. Guilt is rather concerned with *one’s self as an actual or potential wrongdoer*.

### Is Perceived Responsibility a Sufficient Cognitive Component of Guilt?

The self-evaluation of *responsible harmfulness* is a twofold assumption—a self-evaluation of harmfulness *plus* a self-ascription of responsibility. Responsibility is in itself a neutral notion. Responsibility judgments assess whether one caused something, good or bad as it may be, and intended to cause it or at least could prevent it from occurring. Assignment of responsibility and assignment of guilt should be kept distinct (e.g., Shaver, 1985).

One can acknowledge responsibility for a behavior, personal attitude or attribute *without evaluating the latter as harmful*. Thus, perceived responsibility, albeit necessary, is an insufficient component of guilt. Another necessary requirement is the self-evaluation of (potential or actual) harmfulness.

As already pointed out, an evaluation of responsible harmfulness is a moral evaluation. Therefore guilt, being concerned with one’s self as an actual or potential wrongdoer, implies a blow to one’s moral self-image.

A blow to one’s moral *image* is *insufficient* for experiencing guilt. Although one’s moral standards are largely a sociocultural product, and “different moral orders favour different moralities... in their members“ (Benson, 2001, p. 231), others’ evaluation and one’s own self-evaluation may diverge. We possess “'a moral compass' which enables us to know when to turn towards our own feelings and when towards those of other people for guidance in the making of moral choices” (Benson, 2001, p. 131). Thus, one may even acknowledge to “deserve” others’ blame (according to their standards) without sharing in their negative judgment of harmfulness because one evaluates one’s own behavior or attitudes in terms of different standards. In these cases, one will suffer a blow to one’s own moral *image*, but *not* to one’s moral *self-image*.

According to Higgins (1987), different kinds of discrepancy between one’s self-concept and one’s self-guides qualify different kinds of unpleasant emotions. Following self-discrepancy theory, we suggest that guilt is elicited by a discrepancy between the actual and the *ought self*, which prescribes how one should be and behave according to one’s own moral standards. When one feels guilty, one’s moral self-image gets (more or less temporarily) worsened.

In other words, the negative self-evaluation implied in guilt *involves the (moral) self*, and is not confined to one’s specific behavior. In a sense, we agree with Sabini and Silver (1997) when they claim that a “strong” feeling of guilt should involve the self; otherwise guilt is an “anemic” feeling. What we disagree with is the equation of “involving the self” with “involving shame”.

### Is Perceived Non-Responsibility a Necessary Cognitive Component of Shame?

Unlike guilt, shame can be experienced when one’s self-evaluation concerns a mere lack of power. But this doesn’t necessarily imply that one should view oneself as *not* responsible for one’s own faults. Many examples can be found of felt shame concerning a responsible wrongdoing. A child who consciously disobeys his parents, a student who neglects her homework, a soldier who cowardly tries to flee the battle may feel ashamed rather than guilty. But, how is it possible to experience shame in such instances, rather than a misnamed guilty feeling?

What makes the difference between shame and guilt is not the objective kind of fault, but how this fault is represented, and where one’s attention is focused. One can feel shame for a wrongdoing as long as one *does not consider responsibility* issues, and focuses on one’s own *inadequacy* with respect to one’s ideal self. That is, one compares one’s actual self with one’s ideal standard, and finds it is sub-standard.

Higgins (1987) also suggests that shame reflects an actual versus *ideal* self-discrepancy, independent of responsibility issues. However, he states that shame involves “feeling that one has been lowered in the esteem of others because one has disappointed their hopes and wishes for one” (p. 323). We view this condition as both *insufficient* for feeling ashamed (because a lowered *self*-esteem should also be involved) and *unnecessary* (because one may be certain that nobody will ever know about one’s fault, and still feel ashamed).

While acknowledging that the ideal self (as well as the ought self) is socially constructed (e.g., Argyle, 2017) and, developmentally speaking, others’ values and expectations play an important role in shame (e.g., Ferguson, Stegge, & Damhuis, 1991), we suggest that the internalization of social standards involves their possible transformation. Parental values, for instance, may be substantially modified, and even rejected (e.g., Grusec & Goodnow, 1994). In any case, for disappointment of others’ expectations to induce shame in an individual, (s)he should *share* those expectations. So, in any case, the ashamed one has disappointed one’s own expectations for oneself.

The domain of self-esteem involved in shame is a *nonmoral* one, *even when a moral fault is at stake*. No doubt, a soldier who cowardly tries to flee the battle can feel guilty for such a responsible misdeed, suffer a blow to her moral self-image, and blame herself for being “evil”. However it is also possible that she feels *ashamed*, if she remains focused on the discrepancy between her actual self and her ideal self-image of a “brave soldier”. Here, she suffers a blow to her *nonmoral* or “aesthetic” self-image (Sabini & Silver, 1998), viewing herself as “ugly”, defective with respect to her ideal self.

This view of shame offers the remarkable advantage of explaining how it is possible to feel ashamed both of a physical handicap (which involves no personal responsibility) and of a responsible misdeed. In both cases, one compares one’s actual self with one’s ideal self, and is disappointed by the discrepancy between the two. Shame is a kind of *disappointment* concerning the self.

This view of shame can also account for the frequent association of this emotion either with depressive reactions (e.g., Tangney & Tracy, 2012) or with guilt (e.g., Ferguson & Crowley, 1997; Harder, 1995), depending on the outcomes of a further causal search. In fact, as already mentioned, once the perceived fault is attributed to an internal inadequacy, one may trace back the latter to uncontrollable and stable causes, and in these cases depressive reactions, such as helplessness and hopelessness, are likely to ensue; if, conversely, the inadequacy is attributed to unstable and controllable causes, and responsibility implications come into focus, then shame will change into guilt, or “coexist” with guilt—which probably implies recurrent shifts from a mere disappointment in, and dislike of, oneself to guilty self-reproach, depending on where attention is focused.

### Guilt and Shame Concern Different Domains of Self-Esteem

From Freud (1923/1961) on, a distinction has been proposed between the “superego”, or moral conscience or ought self, and the ideal self, concerning one’s wishes and aspirations about oneself (e.g., Higgins, 1987; Kohut, 1977; Piers & Singer, 1971). Self-esteem^vi^ can as well be distinguished into “moral” and “nonmoral” or “aesthetic” (Sabini & Silver, 1998). The negative self-evaluations associated with guilt and shame both involve the self and imply a lowered self-esteem, but concern two distinct domains: the moral domain in guilt, and the aesthetic domain in shame. In a sense it is true that “shame and guilt are not equally ‘moral’ emotions” (Tangney et al., 2007, p. 349): guilt is moral whereas shame is *not* moral. However, this does not mean that shame is morally reprehensible. It is simply *non*moral or amoral, because it implies an aesthetic perspective, even with respect to moral faults. Whereas guilty people view themselves as “evil”, and reproach themselves, ashamed people view themselves as (physically, intellectually or morally) “ugly”, and dislike themselves.

A lowered self-esteem, either in the moral or in the aesthetic domain, doesn’t *necessarily* imply a *global* negative self-view. The latter, although often considered typical of shame, is not necessarily involved in it. For example, going back to our ashamed soldier, her ideal self may include not only the standard of a “brave soldier” but also those of a “considerate friend” or a “loving parent”, which may remain untouched (e.g., Deonna et al., 2012). Moreover, a lowered self-esteem doesn’t necessarily imply stable and uncontrollable self-attributions. Whereas the latter are incompatible with guilt, they are only *compatible* with shame, but not necessarily involved in it. Our ashamed soldier may believe that she is utterly unable to keep up with her ideal standard of a brave soldier because cowardice is something she can’t help, like blindness or palsy. At this point, many dysfunctional consequences that have been typically attributed to shame may ensue—such as depressive symptoms, escape and/or denial, as well as a *stable* low self-esteem. But she can also believe that something can be done, and commit herself to become the person she aspires to be.

## Concluding Remarks

Shame and guilt are unpleasant emotions implying a negative self-evaluation against one’s own standards; both of them can be experienced either publicly or privately; both can be elicited by the same kind of fault; both can trigger either self-defensive or reparative action tendencies; both can have either adaptive or maladaptive implications; and both can involve the self. What are, then, the differences between these emotions?

Shame is an unpleasant emotion implying a *self-evaluation of inadequacy to meet the standards of one’s ideal self*. The self-attributed inadequacy may or may not imply a global negative self-view. Moreover, it may or may not be perceived as stable and uncontrollable. *Only if* it is perceived as uncontrollable and stable, shame will be associated with helplessness and hopelessness. Ashamed people may regard themselves as either responsible or non-responsible for a fault, but in any case, *when experiencing pure shame*, *they are not considering responsibility issues*. As long as one focuses on one’s own inadequacy with respect to the ideal self, one can feel shame (rather than guilt) for a wrongdoing. In fact, although a self-evaluation of inadequacy may concern moral attributes – that is, harmful attributes for which one may view oneself as responsible – ashamed people are only considering the disappointing discrepancy between their ideal (good) self and their actual (not so good) self. Of course this discrepancy implies a “good/bad” dichotomy, but the meaning of “good/bad” is not necessarily *moral*. “Good/bad” may mean not only virtuous/wicked, but also competent/incompetent, beautiful/ugly, disappointing/satisfactory, and so on. Shame is a nonmoral emotion, meaning that it involves the nonmoral or “aesthetic” facet of one’s self-esteem, which is concerned with the self’s adequacy with respect to its own wishes or aspirations.

Guilt is an unpleasant emotion implying a *negative self-evaluation against one’s moral standards,* that is, the standards concerning those behaviors, goals, beliefs or traits for which one regards oneself as responsible. The evaluation is negative in that such behaviors, goals, etc. are viewed as harmful. Therefore, guilt implies a self-evaluation *of responsible harmfulness*, that is, wrongfulness. The wrongdoing can be either actual or potential, that is, a possible consequence of personal traits and dispositions—provided the person views such traits as modifiable through effort (thereby feeling responsible for not trying to modify them). Therefore the self is involved in guilt, in that the fault can be ascribed not only to one’s behavior but also to the self. When one feels guilty, one’s self-image gets (more or less temporarily) worsened. However, in guilt it is the moral facet of one’s self-esteem – the facet concerned with the responsible harmfulness or beneficialness of the self’s behavior, attitudes, and dispositions – that suffers a blow.

In our view, the distinguishing criteria we have suggested allow to account for both the similarities and the differences between shame and guilt, as well as to clarify the most problematic cases. They explain how a moral fault can elicit shame rather than guilt; or, conversely, how a flaw of character can elicit guilt rather than shame. We have also questioned the widespread view of shame as an ugly and maladaptive emotion, versus guilt as a prosocial and adaptive one. Either emotion (and probably any emotion) can be adaptive or maladaptive depending on contextual factors and the regulation strategies used (e.g., Barrett, 1995; Ferguson & Stegge, 1998). Dysfunctionality is not intrinsic to the emotion, but depends on the emotion regulation skills of the experiencing person (e.g., Gross, 1998; Gupta, Rosenthal, Mancini, Chaevens, & Lynch, 2008).
